# Understanding Current Instabilities in Conductive Atomic Force Microscopy

**DOI:** 10.3390/ma12030459

**Published:** 2019-02-01

**Authors:** Lanlan Jiang, Jonas Weber, Francesco Maria Puglisi, Paolo Pavan, Luca Larcher, Werner Frammelsberger, Guenther Benstetter, Mario Lanza

**Affiliations:** 1 Institute of Functional Nano and Soft Materials, Collaborative Innovation Center of Suzhou Nanoscience & Technology, Soochow University, Suzhou 215123, China; lanlan20151992@163.com (L.J.); jonas.weber1991@gmail.com (J.W.); 2 Department of Electrical Engineering, Media Technology and Computer Science, Deggendorf Institute of Technology, 94469 Deggendorf, Germany; guenther.benstetter@th-deggendorf.de; 3 Dipartimento di Ingegneria “Enzo Ferrari”, Università degli Studi di Modena e Reggio Emilia, 41125 Modena, Italy; francescomaria.puglisi@unimore.it (F.M.P.); paolo.pavan@unimore.it (P.P.); 4 Dipartimento di Scienze e Metodi dell’ Ingegneria, Università di Modena e Reggio Emilia, 42122 Reggio Emilia, Italy; luca.larcher@unimore.it; 5 Department of Mechanical Engineering and Mechatronics, Deggendorf Institute of Technology, 94469 Deggendorf, Germany; werner.frammelsberger@th-deg.de

**Keywords:** CAFM, tip degradation, tunneling current, water meniscus, modeling

## Abstract

Conductive atomic force microscopy (CAFM) is one of the most powerful techniques in studying the electrical properties of various materials at the nanoscale. However, understanding current fluctuations within one study (due to degradation of the probe tips) and from one study to another (due to the use of probe tips with different characteristics), are still two major problems that may drive CAFM researchers to extract wrong conclusions. In this manuscript, these two issues are statistically analyzed by collecting experimental CAFM data and processing them using two different computational models. Our study indicates that: (i) before their complete degradation, CAFM tips show a stable state with degraded conductance, which is difficult to detect and it requires CAFM tip conductivity characterization before and after the CAFM experiments; and (ii) CAFM tips with low spring constants may unavoidably lead to the presence of a ~1.2 nm thick water film at the tip/sample junction, even if the maximum contact force allowed by the setup is applied. These two phenomena can easily drive CAFM users to overestimate the properties of the samples under test (e.g., oxide thickness). Our study can help researchers to better understand the current shifts that were observed during their CAFM experiments, as well as which probe tip to use and how it degrades. Ultimately, this work may contribute to enhancing the reliability of CAFM investigations.

## 1. Introduction

Since its invention in 1993 by Murrel et al. [1], conductive atomic force microscopy (CAFM) has experienced continuous developments, and nowadays it has become one of the most powerful tools in studying the electrical properties of materials and devices at the nanoscale [2,3]. CAFM uses an ultra-sharp and conductive tip, which is typically made of Si and coated with a thin (<20 nm) metallic layer, located at the end of a cantilever that is put in contact with the sample under test. The radius at the apex (*R_TIP_*) of metal-coated Si tips ranges from 2 nm to 50 nm, and it allows collecting the currents flowing across single locations of the sample, whose effective areas (namely *A_eff_*) can range between 1 nm^2^ and 800 nm^2^ [2,4]. One of the main advantages of CAFM is that it allows collecting topographic and current information about the samples simultaneously and independently. This is possible because the topographic information is collected using an optical system (i.e., the deflection of the cantilever, when scanning the sample, is detected using a laser and a photodiode), and the electrical information is collected using a preamplifier that is connected to the CAFM tip [2]. This technique was initially designed to analyze the tunneling current across thin dielectric films [1,2], which is still one of its main applications, but it rapidly spread to other fields of science, such as piezotronics and nanochemistry (among many others) [2].

Despite the tremendous advantages of CAFM in terms of lateral resolution and topography-current correlation, its use still presents a major problem that is still not fully understood: the large current variations observed during the experiments, not only during a single study, but also from one CAFM report to another. The two main sources of current variability are: (i) the degradation of the metallic coating of the CAFM tips [4]. This problem might be minimized (up to a certain degree) by using stable CAFM tips (e.g., solid metallic tips [5]). However, these tips are not only much more expensive than standard metal-coated Si tips, but they also may produce a reduction of the lateral resolution of the technique due to their larger *R_TIP_*. For this reason, metal-coated Si tips are still being used, despite their faster degradation. Manufacturers of solid highly-doped diamond tips claimed sub-nanometer lateral resolution [6], but these tips are so stiff that they can easily damage almost every sample. Another possibility is to use metal-coated Si tips protected with a thin layer of graphene (which does not increase *R_TIP_*), but this solution is still in an experimental stage [7,8,9]. Therefore, understanding the degradation process of the metallic coating of the CAFM tips is essential. (ii) the presence of water molecules (H_2_O) between the tip and the sample [10,11]. Although few CAFMs offer the possibility of measuring in vacuum [12,13], most CAFM studies are still conducted in air, meaning that this is a problem that affects most CAFM users. Moreover, not necessarily the vacuum levels that are provided by commercial setups (10^−4^–10^−5^ torr) may be able to completely remove all the H_2_O molecules at the tip/sample interface, which might require heating the sample above 100 °C [14]. Therefore, understanding under which circumstances H_2_O molecules are present at the tip/sample junction is essential for a correct interpretation of the electrical signals collected via CAFM. 

In this work, the current variations observed in CAFM measurements are studied via computational methods. First, we observe that the degradation of the CAFM tips occurs in two steps: initially, the contact resistance increases by 2–3 orders of magnitude, and later the tips completely lose their conductivity. Second, we detected that some CAFM tips cannot provide a good contact during spectroscopic current vs. voltage (I-V) curves, even if the maximum contact force (*F_C_*) allowed by the system is applied. It is very important to detect when these phenomena take place, otherwise the interpretation of the current signals that are detected by the CAFM would be erroneous.

## 2. Experimental

**Figure 1:**
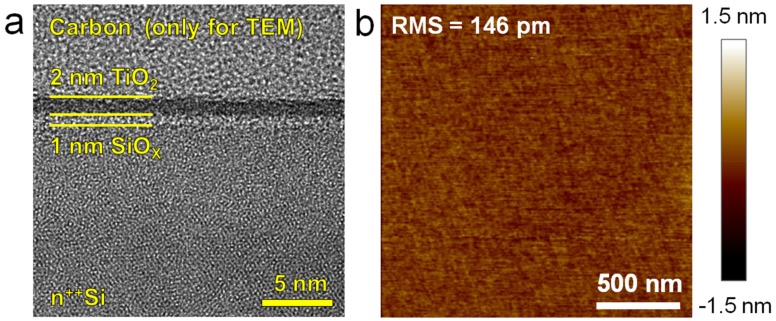
(**a**) Cross sectional transmission electron microscopy (TEM) image and (**b**) atomic force microscopy (AFM) topographic map of a TiO_2_/SiO_x_/n^++^Si sample.

The samples that were used in this study consisted of 2 nm TiO_2_ grown by plasma enhanced atomic layer deposition system (PEALD, Savannah, Cambridge Nanotech, Cambridge, UK) on highly doped n-type Si (n^++^Si) wafers with a resistivity of 0.008–0.02 Ω·cm^−1^. These samples are quite standard and they match well with those used in previous CAFM studies [15,16]. Tetrakis (dimethylamido) titanium [Ti(NMe_2_)_4_] and oxygen were used as Ti and O sources, respectively. The temperature that was used during the deposition process was 200 °C and the TiO_2_ film has been grown at a constant deposition rate of 0.51 Å/cycle (i.e., the growth of 2 nm required 40 cycles). An ALD pulse consisted of exposure to Ti(NMe_2_)_4_ for 0.1 s plus waiting 10 s more. The plasma process used an oxygen flow of 30 sccm for 20 s, a power of 300 W, and a waiting time of 5 s. Before the TiO_2_ deposition, the wafers were rinsed in hydrofluoric acid (HF) with a concentration of 4% for 2 min in order to remove the native SiO_x_ layer on the surface. Cross sectional transmission electron microscopy (TEM) images confirmed the correct thickness of the TiO_2_ layer grown (see Figure 1), and they also revealed the formation of a ~1 nm thick interfacial SiO_x_ layer between the TiO_2_ and the Si substrate. This layer is unavoidably formed due to the interaction of O atoms from the TiO_2_ with the Si substrate [17,18]. Topographic maps that were collected via CAFM (without bias) indicate that the roughness of the TiO_2_ surface (<200 pm) is similar to that of the as-received Si wafers (<200 pm, see Reference [19]), indicating that the TiO_2_ coating is conformal and of high quality (see Figure 1b). The TEM equipment used was a JEM-2100 from JEOL (Akishima, Tokyo, Japan), and the CAFM was a Digital Instruments Dimension 3100 from Veeco (Plainview, NY, USA). All of the CAFM measurements were carried out in normal air atmosphere.

Two types of CAFM tips, named SCM-PIT and PFTUNA, have been used in this study. Both of them are made of a non-conductive bulk material (Si and SiN) coated with a 20 nm thick film of PtIr (an alloy containing 95% Pt and 5% Ir). The SCM-PIT tip has a slightly smaller *R_TIP_* (20 nm typical, 25 nm maximum) as compared to the PFTUNA tip (25 nm typical, 35 nm maximum). The only remarkable difference between the two types of CAFM tips is the spring constant (*k_c_*), 2.8 N/m for the SCM-PIT tip and 0.4 N/m for the PFTUNA tip (typical values). Sequences of I-V curves have been recorded at several different locations on the surface of the TiO_2_/SiO_x_/n^++^Si sample, using both types of tips. In order to avoid point-to-point interferences, the distance between the two I-V curves was >1 µm. During the collection of the I-V curves, the speed of the ramped voltage stress (RVS) was 0.1 Hz and the current was limited to a range between −100 pA and 100 pA. In order to avoid local anodic oxidation, positive bias was applied to the CAFM tip, while the sample holder was grounded [20]. The deflection setpoint (DS) used was 4 V for both tips; this results in a contact force of ~151 nN for the PFTUNA tip and ~526 nN for the SCM-PIT tip. We intentionally used a higher force (by setting up DS = 4V) than that used in other works (which normally use DS = 0 V) in order to induce a good tip/sample contact [21]. It is worth noting that, as we are collecting only spectroscopic measurements and negligible lateral frictions apply, the use of a high contact force should not provoke premature CAFM tip degradation. Despite being very useful for the characterization of several nanomaterials, current/resistance maps [22,23,24] are not used in this study, because that exposes the tip to high lateral and nearly uncontrollable frictions.

## 3. Results and Discussion

**Figure 2:**
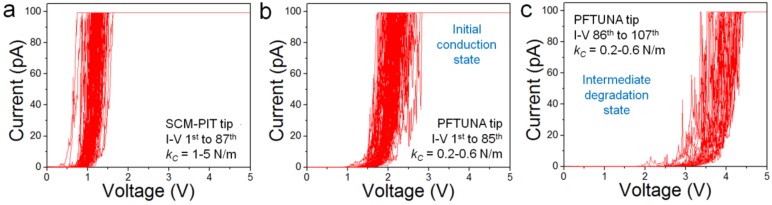
(**a**) 87 I-V curves collected at different locations of the TiO_2_/SiO_x_/n^++^Si sample using a SCM-PIT probe. (**b**) Initial 85 I-V curves and (**c**) final 22 I-V curves collected at different locations of the same sample using a PFTUNA probe (the same for both panels). These I-V curves were obtained under atmospheric environment (normal air). The X-axis represents the tip voltage, while the sample holder was grounded.

For the SCM-PIT tip, 87 I-V curves were recorded by applying RVS from 0 V to a maximum voltage (*V_MAX_*) of 5 V. The obtained results are depicted in Figure 2a. The onset potential (*V_ON_*) of the I-V curves, defined as the minimum voltage at which the current reaches 10 pA, ranged from 0.49 V to 1.35 V. After these 87 I-V curves, the SCM-PIT tip still kept its initial conductivity, and no signs of degradation were detected. For the PFTUNA tip, 107 I-V curves were also recorded using RVS from 0 V to *V_MAX_* = 5 V. However, for this PFTUNA tip, two groups of I-V curves can be distinguished: the initial 85 I-V curves show *V_ON_* between 1.48 V and 2.10 V (see Figure 2b), and the final 22 I-V curves show *V_ON_* between 2.81 V and 3.80 V (see Figure 2c). The transition from one group to another was sharp, and after these 107 I-V curves, the tip completely lost its conductivity, i.e., no currents above the noise level were observed, even when applying the maximum bias that is allowed by the CAFM (which is 10 V). 

### 3.1. Degradation of the CAFM Tip

By comparing Figure 2b and Figure 2c, it can be concluded that the PFTUNA CAFM tip has been degraded. In Reference [4], a complete study regarding the degradation of different CAFM tips has been presented, and it was concluded that the conductivity of the tips could be degraded by metallic varnish melting, by tip apex removal, and by the adhesion of particles. However, the kinetics of this degradation process are not presented, i.e., the differences between the 1st and 20th current scans are compared, but the way in which the current signal decays is not analyzed. Furthermore, the degradation process during lateral scans (analyzed in Reference [4]) may not be the same as the one taking place during spectroscopic I-V curves (this work). On one hand, it is expected that lateral scans consume the bulk of the tips faster due to high lateral frictions [4], and they also attach more impurities at the apex collected during the scan, while these phenomena may be minimized in spectroscopic I-V curves. On the other hand, long sequences of spectroscopic I-V curves may produce faster metallic varnish melting due to the prolonged circulation of high currents [8]. From Figure 2b,c, it can be concluded that the tip does not completely lose its conductivity abruptly, but there is an intermediate state in which stable measurements can be achieved (Figure 2c), before completely losing the conductivity. However, in this state, the contact resistance of the CAFM tips is much larger (see the larger *V_ON_*), which is most probably due to the wearing of the metallic varnish after a long sequence of I-V curves. Therefore, researchers need to be very careful, as measuring in this intermediate state may lead to incorrect conclusions about the sample (e.g., overestimate the oxide thickness).

In order to further investigate and quantify the degradation of the CAFM tips, we conduct another experiment consisting of placing a metal-coated Si tip on the surface of a metallic substrate (a 300 nm SiO_2_/Si wafer coated with 100 nm Pt via ALD, with the Pt film connected to the CAFM plate via silver paint), and collect sequences of I-V curves using an external source meter (model Keithley 6430, Keithley Instruments, Cleveland, OH, USA) [25,26,27]. The use of a pure metallic junction between tip and sample allows monitoring the performance of the metallic coating of the CAFM tip, without the need of considering the tunneling current across the TiO_2_/SiO_x_/n^++^Si sample. The use of a source meter enables measuring larger currents, and therefore allows quantifying the maximum threshold current at which tip degradation takes place (*I_MAX_*).

**Figure 3:**
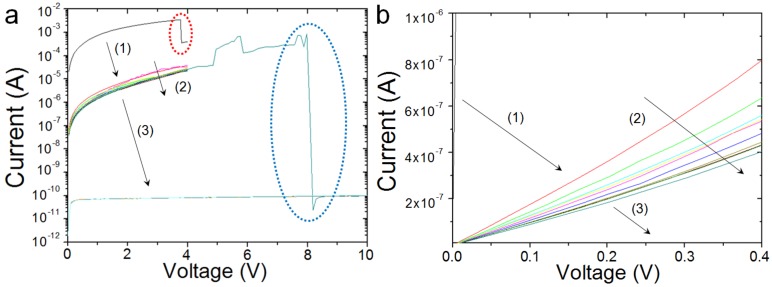
(**a**) Sequences of I-V curves collected at the same position on the surface of a 100 nm Pt/300 nm SiO_2_/Si sample, using a conductive atomic force microscopy (CAFM) connected to an external Keithley 6430 source meter (the Pt film was connected to the CAFM plate using silver paint). The red dashed circle indicates the initial partial degradation of the CAFM tip, followed by an intermediate state (2); the blue dashed circle shows the complete degradation of the CAFM tip. (**b**) The same data is shown in different scales.

The results are displayed in Figure 3. Both of the plots show the same data, but in different scales. As it can be observed, the first I-V curve shows a perfect linear shape, followed by a sudden current decrease at *I_MAX_* = 3.5 mA (see red circle in Figure 3a). From the linear region of the I-V curve, the initial contact resistance can be calculated as R = V/I = 4.54 kΩ. This value is consistent with that provided by the manufacturer of the CAFM tips. After the sudden current decrease, the CAFM tip still shows nearly stable linear behavior, but in this case the contact resistance is 5.88 MΩ. This is consistent with the stable resistive state observed in Figure 2c and it indicates that the degradation of the tip takes place in two steps (most probably the tip coating first narrows before completely melting). Again, this intermediate state may easily drive the CAFM users to wrong conclusions about the materials under investigation. Finally, if the stress proceeds, the complete degradation of the CAFM tips takes place at around 8 V and *I_MAX_* = 510 µA (see blue circle in Figure 3a). 

### 3.2. Presence of H_2_O at the Tip/Sample Junction

In order to discover the differences of the electrical signals that were collected with each type of CAFM tips, the I-V curves have been analyzed using different computational models. In the past, the equations of different tunneling models, including Direct Tunneling [28], Fowler-Nordheim Tunneling [29], Poole-Frenkel [30], and even combinations of a few of them [16], have been used to study the currents across thin dielectrics. These methods have been proved to be very useful when studying single layer dielectrics (i.e., SiO_2_, HfO_2_ [31,32]). In our case, given the complexity of the TiO_2_/SiO_x_ bilayer system, a professional multilevel computational platform, named Ginestra^TM^ (Version, MDLab s.r.l., Reggio Emilia, Italy), has been used [33,34]. This platform is specifically designed for an accurate simulation of charge transport and degradation in dielectric stacks, and it includes a self-consistent description of many transport mechanisms, such as DT (Direct Tunneling), FNT (Fowler-Nordheim Tunneling), and multiphonon trap-assisted tunneling. Ginestra^TM^ allows full three-dimensional (3D) simulations of complex multilayer structures taking advantage of material-specific parameters and the presence of defects. The latter also considers the effects of localized power dissipation (at defects) on the local temperature, as well as the local electric field distortions induced by charge trapped at defect sites. 

**Figure 4:**
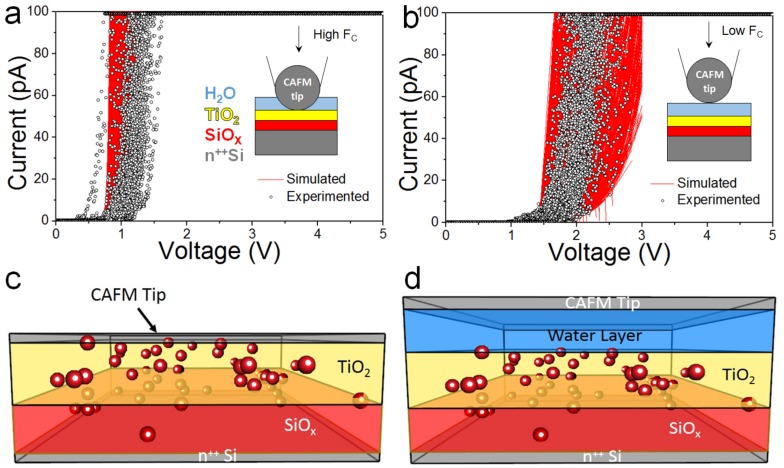
Experimental I-V curves recorded at different locations of the TiO_2_/SiO_x_/n^++^Si samples using (**a**) a SCM-PIT and (**b**) a PFTUNA tip, and their corresponding fittings using Ginestra^TM^ software. The experimental curves correspond to those in Figure 2a,b. Several simulated curves are displayed to reproduce intrinsic variability of the experiments (density of traps, thickness of each layer, and tip/sample contact area). The inset images in (**a**) and (**b**) are the schematics of each configuration. (**c**) and (**d**) show the schematics provided by Ginestra^TM^ software for the SCM-PIT/TiO_2_/SiO_x_/n^++^Si and the PFTUNA/H_2_O/TiO_2_/SiO_x_/n^++^Si structures. Red spheres in the TiO_2_ and SiO_x_ layers represent intrinsic defects.

Figure 4a shows the fitting of the 87 I-V curves that were obtained using SCM-PIT tips. To do this fitting, we considered a Pt/TiO_2_/SiO_x_/n^++^Si structure with a 2 nm thick TiO_2_ layer, a 1 nm thick SiO_x_ layer, and *A_eff_* = 100 nm^2^. The material parameters (electron affinity, *φ*, bandgap, *E_g_*, and dielectric permittivity, *ε*) used for TiO_2_ and SiO_x_ are $\varphi_{TiO_{2}}$ = 3.55 eV, $E_{g,TiO_{2}}$ = 3 eV, $\varepsilon_{TiO_{2}}$ = 60, $\varphi_{SiO_{x}}$ = 0.95 eV, $E_{g,SiO_{x}}$ = 8.9 eV, and $\varepsilon_{SiO_{x}}$ = 6.6. Oxygen vacancy defects were also included in both layers, with a density of defects of 5 × 10^19^ cm^−3^. The schematic of the structure, as provided by the Ginestra^TM^ software, is displayed in Figure 4c. In Figure 4a, 300 randomized devices were simulated to reproduce experimental variability in the simulated I-V curves. These simulated curves include the effect of the random defect position in space and energy and they are based on oxide thickness variations (±0.3 nm for both layers), and possible contact area deviations around the average value (from 8 × 8 nm^2^ to 12 × 12 nm^2^, with an average value *A_eff_* = 10 × 10 nm^2^ = 100 nm^2^). Overall, the simulated I-V curves can fit the experimental ones well. 

In the next step, we try to fit the I-V curves that were obtained using PFTUNA tips. As mentioned, these two types of tips only exhibit one remarkable difference: the *k_c_* of the SCM-PIT tips is seven times lower than the *k_c_* of the PFTUNA tips. It should be highlighted that a change in *k_c_* only modifies the value of *A_eff_* in the tip/sample system [35]. The relationship between them is described in Equations (1) and (2):

(1) $$A_{eff}=A_{c}=\pi r_{c}^{2}=\pi {\left(\frac{F_{c}R_{tip}}{K}\right)}^{\frac{2}{3}}$$

(2) $$\frac{1}{K}=\frac{3}{4}\left(\frac{1-\nu_{1}^{2}}{E_{1}}+\frac{1-\nu_{2}^{2}}{E_{2}}\right)$$

where, *A_c_* is the contact area, *r_c_* is the radius of the contact area, ν_1_ and ν_2_ are the Poisson ratio of the tip and the sample (respectively), *E*_1_ and *E*_2_ are the elasticity modulus of the tip and the sample (respectively), and *F_c_* = *k_c_* × *δ_c_*, with *δ_c_* being the deflection of the cantilever. It should be highlighted that, strictly speaking, *A_eff_* does not equal *A_c_*, as the electric field may be confined at some specific locations of *A_c_* (in such case *A_eff_* < *A_c_*) or spread to surrounding areas (in such case *A_eff_* > *A_c_*) [2]. However, in our experiment, this approximation is reasonable because: (i) the sample being measured is an insulator, which limits electrical field spreading and (ii) the value of *F_c_* is not very high, which does not produce significant electrical field confinement (very high *F_c_* producing field confinement only appear for tips with *k_c_* > 20 N/m) [2]. 

Therefore, for the Ginestra^TM^ fitting of the I-V curves that were obtained with PFTUNA tips, we used identical parameters to those that were used for SCM-PIT tips, with the only difference of *A_eff_*. The relationship between *A_eff_* for SCM-PIT and PFTUNA tips can be calculated from Equations (1) and (2), and it is *A_eff.SCM-PIT_* = 1.94 × *A_eff.PFTUNA_*. Therefore, as the average value of *A_eff.SCM-PIT_* considered in Figure 4a is 100 nm^2^, the average value of *A_eff_* used to fit the I-V curves that were collected with PFTUNA tips was 29.90 nm^2^. However, while using this procedure, we observe that the simulated I-V curves do not fit the experimental ones (not shown). We calculated the minimum possible value of *A_eff_* by using *R_TIP.MIN_* and *k_c.MIN_*, which results in a ratio of *A_eff.SCM-PIT_* = 5 × *A_eff.PFTUNA_*, but even in this case the experimental I-V curves could not be fitted. Therefore, the smaller currents that were observed for PFTUNA tips (Figure 2b) as compared to the SCM-PIT tips (Figure 2a) are not only related to a decrease of *A_eff_* (due to the lower *k_c_*), but they must be related to other additional factors.

As the properties of the tips are very similar and the sample and CAFM are the same, the only feasible explanation for the large current reduction when using PFTUNA tips is the presence of an ultra-thin nanogap between the CAFM tip and the sample, which may be filled by water molecules because the measurements have been carried out in air atmosphere [11,36]. This may happen due to the lower *k_c_* of PFTUNA tips (as compared to the SCM-PIT tips), which should result in a lower *F_c_*. However, this observation is still very surprising, because we intentionally applied a high *F_c_* ~151 nN by setting DS = 4 V.

In order to find out whether a water film is present at the tip/sample interface, the I-V curves that were collected with PFTUNA tips have been simulated again using exactly the same parameters than those used in Figure 4a, with the only difference of an ultra-thin H_2_O nanogap (1.2 ± 0.1 nm) between the PFTUNA tip and the sample, i.e., Pt/H_2_O/TiO_2_/SiO_x_/n^++^Si, as shown in Figure 4d. The water nanogap is modeled assuming an electron affinity $\varphi_{H_{2}O}$ = 1 eV, a bandgap $E_{g,H_{2}O}$ = 6.9 eV, and a dielectric permittivity $\varepsilon_{H_{2}O}$ = 80 [11,36]. In this case, we observe that the use of H_2_O nanogap leads to a very good fitting of the experimental I-V curves (see Figure 4b). In Figure 4b, the variability of the water layer thickness was also introduced, and in total 300 randomized devices were simulated. The thickness that was calculated for this water nanogap in order to fit the measurements is consistent with that used in previous works for similar samples [11,36]. Therefore, Ginestra^TM^ software simulation is further supporting the idea that, despite applying high DS = 4 V (which produces *F_c_* ~151 nN), the PFTUNA tip was not able to penetrate the H_2_O layer and contact the sample, as shown in the inset image of Figure 4b (compared to the inset image of Figure 4a). 

In order to try to break the water layer, the experiments were repeated by applying the highest DS allowed by this CAFM, which was 10 V; in theory, when using a PFTUNA tip with *k_c_* = 0.4 N/m, DS = 10 V should produce *F_c_* ~378 nN. Surprisingly, in such experiments, the currents also did not change remarkably (minor differences within the variability of those obtained in Figure 2b were observed), and no significant variations were observed when using intermediate DS of 6 V and 8 V. Despite that other authors also observed no significant current differences in I-V curves above a specific threshold DS [21], the fact that a theoretical *F_c_* ~378 nN cannot break the water layer makes us believe that the real *F_C_* applied by the CAFM when using DS = 10 V might not reach such value. In fact, at such high forces, some materials even showed to be scratched by the CAFM tip [37,38]. Most probably, the capillary forces that were derived from the water meniscus at the tip/sample junction add a repulsive force that compensates the ones being applied by the DS [35].

Indeed, this result indicates that PFTUNA tips (*R_TIP_* = 35 nm and *k_c_* = 0.2–0.6 N/m) are not suitable to collect sequences of I-V curves under environmental conditions in this type of sample. However, this does not mean that all previous CAFM works using this setup may be erroneous. Definitely, one cannot get quantitative I-V curves with this type of tips (*k_c_* = 0.2–0.6 N/m) under air atmosphere, even by applying the highest contact force (i.e., DS) allowed by the CAFM, but when analyzing different samples, relative variations may still be meaningful. Moreover, the nanogap detected during spectroscopic I-V curves may not necessarily be present during lateral scans, as the lateral movement of the tip may facilitate pushing away the H_2_O molecules.

## 4. Conclusions

In conclusion, we have presented the characterization of TiO_2_/SiO_x_/n^++^Si samples by collecting >80 I-V curves at different locations via CAFM, using two types of tips that are nearly identical, with the only difference being that one has a *k_c_* = 0.4 N/m and the other *k_c_* = 2.8 N/m. Interestingly, the currents that were collected for the tip with *k_c_* = 0.4 N/m are much lower than expected, and they could not be fitted by a reduction of *A_eff_*. By means of computational calculations, we conclude that the large current reduction is related to the formation of a H_2_O nanogap between the CAFM tip and the sample, due to the lower contact force. Surprisingly, this nanogap could not be removed even when applying the maximum contact force allowed by the equipment (using DS = 10 V). Moreover, we characterize the entire degradation process of the CAFM tips, and we observe that before complete degradation there is an intermediate stable state with higher contact resistance. The presence of the water layer or the partial degradation of the tips are essential issues to consider when analyzing CAFM data, as they may drive the users to wrong interpretations (e.g., claiming wrong *d_ox_* or *V_ON_* values). 

## References

[1] Murrell M.P., Welland M.E., O’Shea S.J., Wong T.M.H., Barnes J.R., McKinnon A.W., Heyns M., Verhaverbeke S. (1993). Spatially resolved electrical measurements of SiO_2_ gate oxides using atomic force microscopy. Appl. Phys. Lett..

[2] Pan C., Shi Y., Hui F., Gutierrez E.G., Lanza M., Lanza M. (2017). History and Status of the CAFM. Conductive Atomic Force Microscopy: Applications in Nanomaterials.

[3] Wu Q., Bayerl A., Porti M., Martínez J.M., Lanza M., Rodriguez R., Velayudhan V., Nafria M., Aymerich X., Gonzalez M.B. (2014). A Conductive AFM Nanoscale Analysis of NBTI and Channel Hot-Carrier Degradation in MOSFETs. IEEE Trans. Electron Devices.

[4] Krause O., Lanza M. (2017). Fabrication and Reliability of Conductive AFM Probes. Conductive Atomic Force Microscopy: Applications in Nanomaterials.

[5] Rocky Mountain Nanotechnology: Technical Data. http://rmnano.com/tech.html.

[6] Imec: Solid Diamond AFM Probes Datasheet. https://www.brukerafmprobes.com/images/product/specPDF/3820.pdf.

[7] Hui F., Chen S., Liang X., Yuan B., Jing X., Shi Y., Lanza M. (2017). Graphene Coated Nanoprobes: A Review. Crystals.

[8] Hui F., Vajha P., Shi Y., Ji Y., Duan H., Padovani A., Larcher L., Li X.R., Xu J.J., Lanza M. (2016). Moving graphene devices from lab to market: Advanced graphene-coated nanoprobes. Nanoscale.

[9] Hui F., Vajha P., Ji Y., Pan C., Gutierrez E.G., Duan H., He P., Ding G., Shi Y., Lanza M. (2017). Variability of graphene devices fabricated using graphene inks: Atomic force microscope tips. Surf. Coat. Technol..

[10] Weeks B.L., Vaughn M.W. (2005). Direct imaging of meniscus formation in atomic force microscopy using environmental scanning electron microscopy. Langmuir.

[11] Kremmer S., Peissl S., Teichert C., Kuchar F., Hofer H. (2003). Modification and characterization of thin silicon gate oxides using conducting atomic force microscopy. Mater. Sci. Eng. B.

[12] Parksystems.com: Park NX-Hivac. www.parksystems.com/index.php/products/small-sample-afm/park-nx-hivac.

[13] Hitachi-hightech.com: Hitachi AFM5300E. https://www.hitachi-hightech.com/global/science/products/microscopes/afm/units/afm5300e.html.

[14] Scientaomicron.com: Fermi DryCool SPM. http://www.scientaomicron.com/en/products/434/1386.

[15] Xiao N., Villena M.A., Yuan B., Chen S., Wang B., Eliáš M., Shi Y., Hui F., Jing X., Scheuerman A. (2017). Resistive random access memory cells with a bilayer TiO_2_/SiO_x_ insulating stack for simultaneous filamentary and distributed resistive switching. Adv. Funct. Mater..

[16] Frammelsberger W., Benstetter G., Kiely J., Stamp R. (2007). C-AFM-based thickness determination of thin and ultra-thin SiO_2_ films by use of different conductive-coated probe tips. Appl. Surf. Sci..

[17] Lanza M., Porti M., Nafría M., Aymerich X., Benstetter G., Lodermeier E., Ranzinger H., Jaschke G., Teichert S., Wilde L. (2011). Conductivity and charge trapping after electrical stress in amorphous and polycristalline Al_2_O_3_ based devices studied with AFM related techniques. IEEE Trans. Nanotechnol..

[18] Lanza M., Porti M., Nafría M., Aymerich X., Benstetter G., Lodermeier E., Ranzinger H., Jaschke G., Teichert S., Wilde L. (2009). Crystallization and silicon diffusion nanoscale effects on the electrical properties of Al_2_O_3_ based devices. Microelectron. Eng..

[19] Shi Y., Ji Y., Sun H., Hui F., Hu J., Wu Y., Fang J., Lin H., Wang J., Duan H. (2015). Nanoscale characterization of PM_2.5_ airborne pollutants reveals high adhesiveness and aggregation capability of soot particles. Nat. Sci. Rep..

[20] Ji Y., Hui F., Shi Y., Han T., Song X., Pan C., Lanza M. (2015). Fabrication of a fast-response and user-friendly environmental chamber for atomic force microscopes. Rev. Sci. Instrum..

[21] Lee G., Yu Y., Lee C., Dean C., Shepard K.L., Kim P., Hone J. (2011). Electron tunneling through atomically flat and ultrathin hexagonal boron nitride. Appl. Phys. Lett..

[22] Houzé F., Meyer R., Schneegans O., Boyer L. (1996). Imaging the local electrical properties of metal surfaces by atomic force microscopy with conducting probes. Appl. Phys. Lett..

[23] Infante I.C., Sánchez F., Laukhin V., del Pino A.P., Fontcuberta J., Bouzehouane K., Fusil S., Barthélémy A. (2006). Functional characterization of SrTiO_3_ tunnel barriers by conducting atomic force microscopy. Appl. Phys. Lett..

[24] Félix L.A., Sirena M., Guzmán L.A.A., Sutter J.G., Vargas S.P., Steren L.B., Bernard R., Trastoy J., Villegas J.E., Briático J. (2012). Structural and electrical characterization of ultra-thin SrTiO_3_ tunnel barriers grown over YBa_2_Cu_3_O_7_ electrodes for the development of high Tc Josephson junctions. Nanotechnology.

[25] Lanza M., Bayerl A., Gao T., Porti M., Nafria M., Jing G., Zhang Y., Liu Z., Duan H. (2013). Graphene-coated Atomic Force Microscope tips for reliable nanoscale electrical characterization. Adv. Mater..

[26] Shi Y., Ji Y., Hui F., Iglesias V., Marc Porti M., Nafria M., Miranda E., Bersuker G., Lanza M. (2014). Elucidating the origin of resistive switching in ultrathin hafnium oxides through high spatial resolution tools. ECS Trans..

[27] Aguilera L., Lanza M., Bayerl A., Porti M., Nafria M., Aymerich X. (2009). Development of a conductive atomic force microscope with a logarithmic current-to-voltage converter for the study of metal oxide semiconductor gate dielectrics reliability. J. Vac. Sci. Technol. B.

[28] Palumbo F., Liang X., Yuan B., Shi Y., Hui F., Villena M.A., Lanza M. (2018). Bimodal dielectric breakdown in electronic devices using chemical vapor deposited hexagonal boron nitride as dielectric. Adv. Electron. Mater..

[29] Xu J., Xu J., Zhang P., Li W., Chen K. (2013). Nanoscale quantification of charge injection and transportation process in Si-nanocrystal based sandwiched structure. Nanoscale.

[30] Bolsée J., Oosterbaan W.D., Lutsen L., Vanderzande D., Manca J. (2011). CAFM on conjugated polymer nanofibers: Capable of assessing one fiber mobility. Org. Electron..

[31] Lanza M., Porti M., Nafria M., Benstetter G., Frammelsberger W., Ranzinger H., Lodermeier E., Jaschke G. (2007). Influence of the manufacturing process on the electrical properties of thin (<4 nm) Hafnium based high-k stacks observed with CAFM. Microelectron. Reliab..

[32] Lanza M., Iglesias V., Porti M., Nafría M., Aymerich X. (2011). Polycrystallization effects on the variability of the electrical properties of high-k dielectrics at the nanoscale. Nanoscale Res. Lett..

[33] Larcher L., Puglisi F.M., Padovani A., Vandelli L., Pavan P. Multiscale modeling of electron-ion interactions for engineering novel electronic devices and materials. Proceedings of the 26th International Workshop on Power and Timing Modeling, Optimization and Simulation (PATMOS).

[34] Padovani A., Larcher L., Puglisi F.M., Pavan P. Multiscale modeling of defect-related phenomena in high-k based logic and memory devices. Proceedings of the 2017 IEEE 24th International Symposium on the Physical and Failure Analysis of Integrated Circuits (IPFA).

[35] Cappella B., Dietler G. (1999). Force-distance curves by atomic force microscopy. Surf. Sci. Rep..

[36] Pirrotta O., Larcher L., Lanza M., Padovani A., Porti M., Nafria M., Bersuker G. (2013). Leakage current through the poly-crystalline HfO_2_: Trap densities at grains and grain boundaries. J. Appl. Phys..

[37] Chen S., Jiang L., Buckwell M., Jing X., Ji Y., Grustan-Gutierrez E., Hui F., Shi Y., Rommel M., Paskaleva A. (2018). On the Limits of Scalpel AFM for the 3D Electrical Characterization of Nanomaterials. Adv. Functional Mater..

[38] Celano U., Hsia F.-C., Vanhaeren D., Paredis K., Nordling T.E.M., Buijnsters J.G., Hantschel T., Vandervorst W. (2018). Mesoscopic physical removal of material using sliding nano-diamond contacts. Sci. Rep..

